# BRI shows stronger association than BMI for MACE in patients with T2DM: insights from the ACCORD study

**DOI:** 10.3389/fnut.2025.1720948

**Published:** 2025-12-12

**Authors:** Maojun Liu, Junyu Pei, Cheng Zeng, Ying Xin, Peiqi Tang, Liang Tang, Xinqun Hu

**Affiliations:** Department of Cardiovascular Medicine, The Second Xiangya Hospital, Central South University, Changsha, Hunan, China

**Keywords:** body roundness index, body mass index, major adverse cardiovascular events, type 2 diabetes mellitus, action to control cardiovascular risk in diabetes

## Abstract

**Objective:**

This study investigated the associations of the body roundness index (BRI) with major adverse cardiovascular events (MACEs, including non-fatal myocardial infarction, non-fatal stroke, and cardiovascular death) and total mortality (TM) in patients with type 2 diabetes mellitus (T2DM) from the ACCORD/ACCORDION cohort.

**Methods:**

During a median follow-up of 8.82 years, a total of 1,802 participants (17.76%) experienced MACEs, and 1,926 participants (18.98%) died from all causes. We used Cox proportional hazards regression models to evaluate the association of BRI and body mass index (BMI) with MACEs and TM. Non-linear dynamics between BRI and these outcomes were probed via restricted cubic spline and smooth curve-fitting, identifying pivotal risk thresholds. Comprehensive subgroup and sensitivity analyses were implemented to validate the consistency of the results.

**Results:**

The findings from multivariate Cox regression models, after adjusting for potential confounders, indicated that elevated BRI levels were a significant risk factor for MACEs and TM. A unit rise in BRI was associated with a 3% heightened risk of MACEs and a 6% increase in TM. Patients in the highest tertile of baseline BRI faced a 1.14 fold and a 1.27 fold elevated risk of MACEs and TM, respectively, relative to those in the lowest tertile. In contrast, the same multivariate regression analysis showed no significant association between baseline BMI quartiles and MACEs. Notably, the associations of BRI with MACEs and TM were comparable to those observed for the waist-to-height ratio (WHtR) and the conicity index. Additionally, our analysis delineated a nonlinear correlation between BRI and both types of mortality, pinpointing inflection points at 4.39 for cardiovascular mortality and 4.24 for TM via a recursive methodology.

**Conclusion:**

An elevated BRI was significantly associated with a higher risk of MACEs and TM in our cohort of patients with T2DM. BRI may serve as a useful indicator for assessing cardiovascular risk in patients with T2DM.

**Clinical trial registration:**

https://clinicaltrials.gov/ct2/show/NCT00000620, NCT00000620

## Introduction

Type 2 diabetes mellitus (T2DM) constitutes a significant global public health issue due to its prevalence and morbidity (1–3). According to a 2021 International Diabetes Federation (IDF) survey, approximately 537 million individuals worldwide suffer from T2DM, and it is projected to reach 783 million by 2045 (4). Cardiovascular disease (CVD) is the leading cause of mortality in patients with T2DM, who face a more than twofold increased risk of premature cardiovascular events compared to non-diabetics (5, 6). Therefore, the accurate assessment of cardiovascular and mortality risk in this population is imperative for developing effective preventive and therapeutic strategies. Obesity, a key pathophysiological factor in T2DM, is recognized widely as a substantial contributor to CVD and is intricately linked with poor cardiovascular outcomes in these patients (7–9). Traditionally, the body mass index (BMI) has been the principal gauge for obesity assessment, extensively investigated for its associations with cardiovascular and all-cause mortality (10, 11). However, BMI does not differentiate between fat and muscle mass, nor does it reflect fat distribution, particularly abdominal or visceral fat, which is more strongly associated with cardiovascular risk (12–14). Given that abdominal fat contributes more to cardiovascular risk than total body weight, alternative indices reflecting body shape and fat distribution have been proposed.

Recent studies have emphasized the important role of metabolic factors in improving cardiovascular risk stratification among patients with diabetes (15). The body roundness index (BRI), first introduced by Thomas et al. in 2013, introduces a updated approach to evaluating body composition. It determines body fat distribution by amalgamating waist circumference and height measurements, thereby offering an accurate assessment of abdominal fat (16). Additionally, evidence indicates that BRI is predictive of the development of metabolic syndrome, insulin resistance, and hypertension (17–19). Moreover, BRI is simple to calculate in clinical practice, requiring only basic anthropometric measurements, making it feasible for large-scale screening and daily application. Recent studies have reported a nonlinear association between BRI and both cardiovascular and all-cause mortality in the general and hypertensive populations in the United States (20, 21). Building on these findings, recent large-scale studies have further extended the prognostic significance of BRI. Zhang et al. (22) demonstrated a U-shaped association between BRI and all-cause mortality in U.S. adults, while Wang et al. (23) found a similar pattern for both all-cause and cardiovascular mortality among individuals with diabetes or prediabetes. Moreover, Li et al. (24) reported that BRI performs comparably or even better than waist-to-hip ratio and waist circumference in identifying cardiometabolic risk factors.

Although previous studies have explored the associations between BRI and all-cause and cardiovascular mortality in general and diabetic populations, its relationship with major adverse cardiovascular events (MACEs) and total mortality (TM) in patients with T2DM at high cardiovascular risk has not been clearly defined. Many individuals with T2DM also have hypertension, dyslipidemia, or obesity, conditions that markedly increase cardiovascular risk. Therefore, evaluating whether BRI is associated with MACEs and TM in this high-risk population is clinically meaningful. In this study, we analyzed data from the Action to Control Cardiovascular Risk in Diabetes (ACCORD) and ACCORDION studies, which provide a large, well-characterized cohort of patients with T2DM and elevated cardiovascular risk. We examined the associations of BRI with MACEs and TM and compared its discriminative performance with other common anthropometric indices, including body mass index (BMI), waist-to-height ratio (WHtR), and the conicity index (CI). Through detailed examination, this research aims to generate new evidence to enhance clinical practice.

## Materials and methods

### Participants and study design

A retrospective analysis was conducted on data sourced from the ACCORD/ACCORDION trial (ClinicalTrials.gov No.: NCT00000620). The detailed design and methodology of the ACCORD trial have been previously described (ACCORD Study Group, Am J Cardiol, 2007; 99(12A): 21i–33i) (25). The ACCORD trial was a randomized, multicenter, 2×2 factorial study. Encompassing 10,251 patients with T2DM with an average age of 62.2 years and a median HbA1c of 8.1%, the study included individuals either already diagnosed with CVD or at high risk of such conditions. Spanning from June 2001 to June 2009 at 77 sites across the United States and Canada, the trial observed subjects for an average of five years. From 2011 to 2014, participants of the ACCORDION trial underwent further monitoring over an average of 3.5 years through clinical visits and telephonic assessments, during which essential medical data were collected, and both MACEs and TM were rigorously assessed by the dedicated Morbidity and Mortality Subcommittee.

### Data collection and outcomes

**Figure 1:**
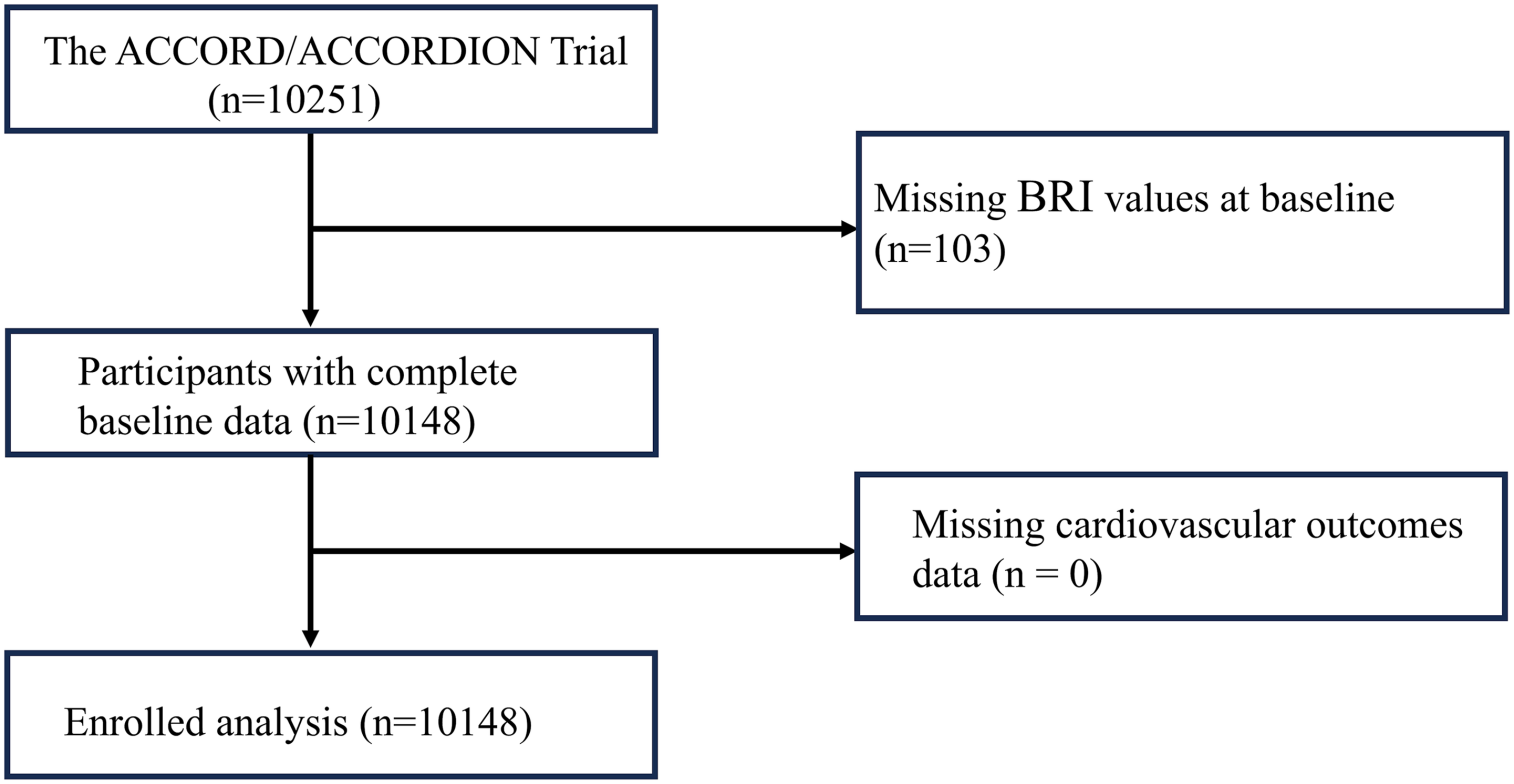
Study participant selection from the Action to Control Cardiovascular Risk in Diabetes (ACCORD)/ACCORD follow-on (ACCORDION) study. BRI, body roundness index.

Our research comes from the ACCORD/ACCORDION trial (ClinicalTrials.gov No.: NCT00000620). The ACCORD/ACCORDION trial collected data on demographic and clinical characteristics, including age, sex, education, ethnicity, treatment and medical history, physical examinations, laboratory tests, and prior medication use. Of the initial 10,251 participants, 103 were excluded due to the absence of baseline BRI data, resulting in 10,148 participants being included in the analysis (Figure 1). The calculations for BRI and BMI were performed as described:

$\mathrm{BRI}=364.2-365.5\times \sqrt{1-\frac{{\left(\mathrm{WC}/2\pi \right)}^{2}}{{\left(0.5\;\text{height}\right)}^{2}}}$

BMI = weight/height^2^ (kg/m^2^).

WHtR = waist circumference (cm)/height (cm)

$\mathrm{CI}=\frac{\text{Waist circumference}\;(m)}{0.019\times \sqrt{\text{Weight}\;(\mathrm{kg})/\text{Height}\;(m)}}$

The primary endpoint was the occurrence of MACEs, which included non-fatal myocardial infarction (MI), non-fatal stroke, and deaths from cardiovascular causes. The secondary endpoint was the incidence of TM. The definition and event ascertainment of cardiovascular outcomes followed those of the ACCORD trial, as previously reported (N Engl J Med, 2011; 364: 818–828) (26).

### Ethics approval and consent to participate

The data for this study were sourced from publicly accessible databases (ACCORD, https://biolincc.nhlbi.nih.gov/studies/accord/). Ethical approval for the ACCORD study was granted by institutional review boards of each clinical site and written informed consent was obtained from all recruited participants (Clinical Trial Registration number: NCT00000620). As this study was based on publicly available de-identified data, no specific ethical approval was required. The dataset is freely accessible and can be used for research and publication purposes.

### Statistical analysis

The statistical analysis was conducted using SPSS 26.0 (IBM, Armonk, NY, USA), R (The R Foundation, Vienna, Austria), and EmpowerStats (X&Y Solutions, Inc., Boston, USA). Baseline characteristics were reported as means ± standard deviations (SD), frequencies and percentages, or medians and interquartile ranges, contingent upon the distribution of the variables. Continuous variables were analyzed using ANOVA or Kruskal-Wallis tests, and categorical variables through Pearson chi-square tests. Kaplan–Meier survival curves illustrated the cumulative risk for MACEs, cardiovascular mortality, and TM, with differences between groups evaluated using the log-rank test. The Cox proportional hazards regression model analyzed the correlation between BRI and the prospective occurrence of MACEs and TM. Initial univariate analysis determined potential confounders by examining the relationship between all collected variables and future MACE occurrences. Selection of covariates was informed by their theoretical justification or clinical significance. In the multivariate analysis, included variables were those that achieved statistical significance in the univariate analysis (*p* < 0.05, Supplementary Table 1), variables of clinical importance strongly linked with MACEs, and those showing an effect size change greater than 10%, irrespective of a univariate *p*-value above 0.05. The analyses utilized an unadjusted model and five incrementally adjusted models to mitigate confounding effects. Model 1 (minimally adjusted) accounted for sex, race, age, education, living situation, CVD history, diabetes duration, prior hypertension and hyperlipidemia, proteinuria, heart failure, smoking, and depression. Model 2 (moderately adjusted) extended Model 1 by including systolic blood pressure (SBP), diastolic blood pressure (DBP), fasting plasma glucose (FPG), hemoglobin A1c (HbA1c), total cholesterol (TC), Triglycerides (TG), low-density lipoprotein cholesterol (LDL-C), high-density lipoprotein cholesterol (HDL-C), estimated glomerular filtration rate (eGFR), and other relevant covariates. Model 3 (comprehensively adjusted) further incorporated medication use, including diuretics, calcium channel blockers (CCBs), beta-blockers, biguanides, meglitinides, thiazolidinediones (TZD), insulins, cholesterol-lowering agents, and aspirin. Collinearity tests were conducted on all adjusted variables included in the analysis. Typically, collinearity is present when the tolerance is less than 0.1 or the variance inflation factor (VIF) exceeds 5. The results showed that the VIF for each variable was less than 5 and the tolerance was greater than 0.1 (Supplementary Table 2), indicating no significant multicollinearity. In our study, there were 103 (1.00%), 4 (0.04%), 101 (0.99%), 4 (0.04%), 46 (0.45%), 46 (0.45%), 53 (0.52%), 22 (0.21%), 55 (0.54%), 55 (0.54%), 55 (0.54%), 54 (0.53%), and 54 (0.53%) participants with missing data for BRI, BMI, WC, height, SBP, DBP, FPG, HbA1c, TC, TG, LDL-C, HDL-C, and GFR, respectively. Multiple imputation was employed to handle missing data, reducing the impact on sample size (27, 28). Sensitivity analyses verified the robustness of the findings. Nonlinear relationships were explored using restricted cubic spline (RCS) and smooth curve fitting; identified inflection points were determined using the recursive method, and the effect size and confidence interval were calculated with a two-segment Cox model.

## Results

### Baseline characteristics of participants

The initial cohort included 10,148 individuals, with males constituting 61.45% and an average age of 62.80 ± 6.65 years. Baseline hematological measurements were recorded as follows: TC at 183.30 ± 41.85 mg/dL; LDL-C at 104.86 ± 33.89 mg/dL; triglycerides (TG) at 190.26 ± 148.59 mg/dL; FPG at 175.20 ± 56.16 mg/dL; HbA1c at 8.30 ± 1.06%; and BRI at 6.20 ± 1.91. These participants were categorized into three tertiles based on their BRI quantiles: Low (Tertile 1, 4.24 ± 0.67), Medium (Tertile 2, 5.96 ± 0.48), and High (Tertile 3, 8.39 ± 1.25) (Table 1). The high BRI cohort predominantly included younger, female, and White participants, with elevated educational attainment and more frequent alcohol usage. Additionally, this group displayed lower diabetes durations, increased instances of solitary living, and higher incidences of depression, heart failure, hypertension, proteinuria, and elevated BMI. Elevated DBP, HR, FPG, TC, and TG levels, along with reduced HDL levels, were observed. These participants were also more likely to administer angiotensin receptor blockers (ARB)/angiotensin-converting enzyme inhibitors (ACEI), CCBs, *β*-blockers, diuretics, thiazolidinediones, insulins, and various other medications, while sulfonylurea usage was relatively minor.

**Table 1:** Baseline characteristics of participants across BRI tertiles.

Characteristics	Overall	Tertile 1 (*n* = 3,382)	Tertile 2 (*n* = 3,380)	Tertile 3 (*n* = 3,386)	*p*-value
BRI	6.20 ± 1.91	4.24 ± 0.67	5.96 ± 0.48	8.39 ± 1.25	<0.001
Age (years)	62.80 ± 6.65	63.02 ± 7.05	63.10 ± 6.58	62.29 ± 6.28	<0.001
Sex, *n* (%)					<0.001
Male	6,236 (61.45%)	2,359 (69.75%)	2,175 (64.35%)	1702 (50.27%)	
Female	3,912 (38.55%)	1,023 (30.25%)	1,205 (35.65%)	1,684 (49.73%)	
Race, *n* (%)					<0.001
White	6,324 (62.32%)	1844 (54.52%)	2,143 (63.40%)	2,337 (69.02%)	
Non-White	3,824 (37.68%)	1,538 (45.48%)	1,237 (36.60%)	1,049 (30.98%)	
Education, *n* (%)					<0.001
Less than high school graduate	1,510 (14.89%)	473 (14.00%)	496 (14.68%)	541 (15.99%)	
High school grad (or GED)	2,674 (26.37%)	853 (25.24%)	928 (27.47%)	893 (26.39%)	
Some college or technical school	3,325 (32.79%)	1,025 (30.33%)	1,116 (33.04%)	1,184 (34.99%)	
College graduate or more	2,632 (25.95%)	1,028 (30.42%)	838 (24.81%)	766 (22.64%)	
CVD History, *n* (%)	3,579 (35.27%)	1,197 (35.39%)	1,207 (35.71%)	1,175 (34.70%)	0.674
Duration of diabetes (years)	10.79 ± 7.59	11.13 ± 7.75	10.69 ± 7.53	10.54 ± 7.46	0.007
Previous hypertension, *n* (%)	7,650 (75.38%)	2,474 (73.15%)	2,527 (74.76%)	2,649 (78.23%)	<0.001
Previous hyperlipidemia, *n* (%)	7,098 (69.94%)	2,336 (69.07%)	2,420 (71.60%)	2,342 (69.17%)	0.037
Proteinuria, *n* (%)	2007 (19.78%)	610 (18.04%)	619 (18.31%)	778 (22.98%)	<0.001
Heart failure, *n* (%)	489 (4.82%)	136 (4.02%)	136 (4.02%)	217 (6.41%)	<0.001
Depression, *n* (%)	2,403 (23.68%)	595 (17.59%)	775 (22.93%)	1,033 (30.53%)	<0.001
Living alone, n (%)	2052 (20.22%)	604 (17.86%)	688 (20.36%)	760 (22.45%)	<0.001
Smoking, *n* (%)					<0.001
Yes	5,917 (58.31%)	1925 (56.92%)	2006 (59.35%)	1986 (58.65%)	
No	4,231 (41.69%)	1,457 (43.08%)	1,374 (40.65%)	1,400 (41.35%)	
Alcohol, *n* (%)					<0.001
Yes	2,424 (23.90%)	902 (26.68%)	869 (25.72%)	653 (19.30%)	
No	7,720 (76.10%)	2,479 (73.32%)	2,510 (74.28%)	2,731 (80.70%)	
BMI (kg/m2)	32.20 ± 5.40	27.21 ± 3.13	31.94 ± 3.17	37.46 ± 3.87	<0.001
SBP (mmHg)	136.37 ± 17.11	136.36 ± 16.97	136.20 ± 16.91	136.56 ± 17.44	0.728
DBP (mmHg)	74.89 ± 10.65	73.97 ± 10.67	74.87 ± 10.48	75.81 ± 10.74	<0.001
Heart rate, bpm	72.66 ± 11.75	71.47 ± 11.51	72.41 ± 11.86	74.09 ± 11.72	<0.001
FPG (mg/dL)	175.20 ± 56.16	173.93 ± 57.98	174.57 ± 55.81	177.09 ± 54.60	<0.001
HbA1C (%)	8.30 ± 1.06	8.28 ± 1.10	8.28 ± 1.03	8.33 ± 1.05	0.012
TC (mg/dL)	183.29 ± 41.85	182.04 ± 41.58	183.10 ± 42.10	184.73 ± 41.85	<0.001
TG (mg/dL)	190.26 ± 148.59	171.86 ± 148.08	195.76 ± 147.01	203.14 ± 148.90	<0.001
VLDL-C (mg/dL)	36.56 ± 24.38	33.12 ± 23.76	37.66 ± 24.71	38.90 ± 24.27	<0.001
LDL-C (mg/dL)	104.86 ± 33.89	105.71 ± 33.37	104.28 ± 34.05	104.60 ± 34.24	0.123
HDL-C (mg/dL)	41.87 ± 11.63	43.21 ± 12.69	41.16 ± 10.98	41.23 ± 11.03	<0.001
eGFR (ml/min/1.73 m2)	91.05 ± 27.18	91.49 ± 28.84	90.49 ± 26.39	91.18 ± 26.22	0.424
Medications, *n* (%)					
Diuretics	3,708 (36.54%)	933 (27.59%)	1,238 (36.63%)	1,537 (45.39%)	<0.001
ARB/ACEI	7,027 (69.25%)	2,199 (65.02%)	2,348 (69.47%)	2,480 (73.24%)	<0.001
CCB	1939 (19.11%)	556 (16.44%)	665 (19.67%)	718 (21.20%)	<0.001
β-Blockers	3,054 (30.17%)	890 (26.41%)	1,059 (31.41%)	1,105 (32.70%)	<0.001
Biguanides	6,489 (63.95%)	2,109 (62.36%)	2,205 (65.26%)	2,175 (64.24%)	0.042
Thiazolidinediones	2,238 (22.06%)	654 (19.34%)	729 (21.57%)	855 (25.25%)	<0.001
Meglitinides	252 (2.48%)	102 (3.02%)	73 (2.16%)	77 (2.27%)	0.049
Sulfonylureas	5,429 (53.50%)	1878 (55.53%)	1834 (54.28%)	1717 (50.71%)	<0.001
Insulins	3,539 (34.87%)	980 (28.98%)	1,174 (34.73%)	1,385 (40.90%)	<0.001
Statins	6,449 (63.78%)	2,112 (62.84%)	2,213 (65.63%)	2,124 (62.88%)	0.024
Aspirin	5,524 (54.68%)	1751 (52.11%)	1897 (56.34%)	1876 (55.57%)	0.001
Cholesterol absorption inhibitors	205 (2.03%)	63 (1.88%)	84 (2.49%)	58 (1.72%)	0.057

Data are showed as mean (standard deviations), median (T1–T3), or as *n* (%). *p* value for the test of the difference across tertiles of BRI were obtained by using the *χ*^2^ test (categorical variables), ANOVA (continuous variables), or Kruskal-Wallis test (nonparametric comparisons).

BRI, body roundness index; BMI, body mass index; GED, general education development; CVD, cardiovascular disease; SBP, systolic blood pressure; DBP, diastolic blood pressure; FPG, fasting plasma glucose; HbA1C, glycated hemoglobin; TC, total cholesterol; TG, triglycerides; LDL-C, low-density lipoprotein cholesterol; eGFR, estimated glomerular filtration rate; ARB, angiotensin receptor blocker; ACEI, angiotensin converting enzyme inhibitors; CCB, calcium channel blockers.

### Association between BRI and MACEs and TM

**Figure 2:**
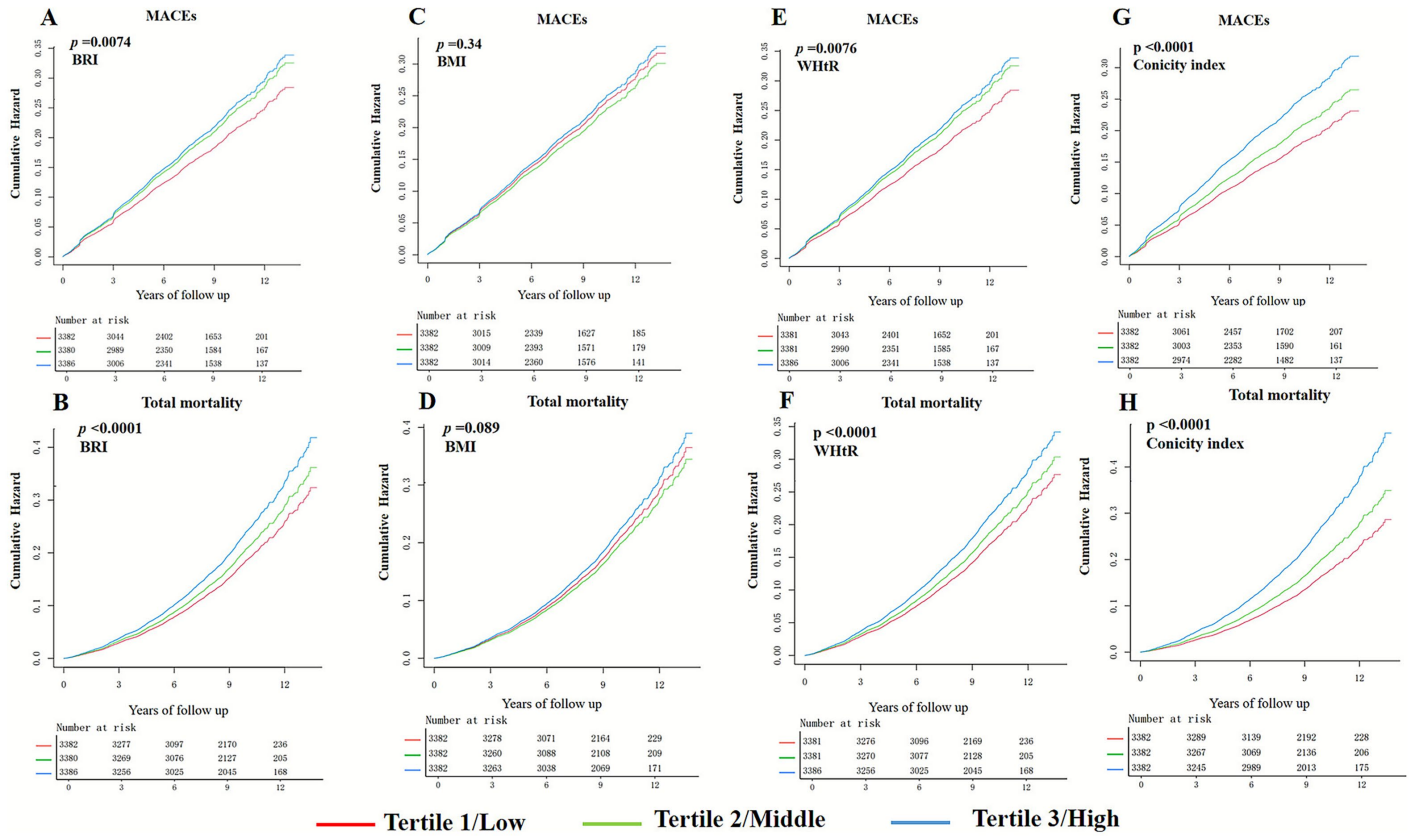
Kaplan–Meier curves for MACEs and total mortality with BRI, BMI, WHtR and conicity index. **(A,B)** BRI; **(C,D)** BMI; **(E,F)** WHtR; **(G,H)** conicity index. BRI, body roundness index; BMI, body mass index; WHtR, waist-to-height ratio; MACEs, major adverse cardiovascular events.

During the median 8.82-year follow-up period, 1,802 participants (17.76%) encountered MACEs, defined as non-fatal myocardial infarction, non-fatal stroke, or cardiovascular death, with 662 instances (6.50%) of cardiovascular mortality reported. In total, 1,926 participants (18.98%) succumbed. Kaplan–Meier analysis assessed the cumulative risk for MACEs and TM, revealing that elevated BRI values correlated with increased risks (log-rank test: MACEs, *p* = 0.0074; TM, *p* < 0.0001). However, an increase in BMI did not reveal a significant association (log-rank test: MACEs, *p* = 0.034; TM, *p* = 0.089) (Figure 2).

Three sequential multivariate regression models were utilized to evaluate the association between BRI and the incidence of outcome events (Table 2). After extensive adjustment for confounding variables, considering BRI as a continuous factor revealed that each unit increase in BRI was linked to a 3% elevation in MACE risk (HR 1.03, 95% CI 1.00–1.06; *p* = 0.0265) and a 6% escalation in TM risk (HR 1.06, 95% CI 1.03–1.08; *p* < 0.0001). In Model 3, BRI persistently showed a strong relationship with elevated cumulative risks of MACEs and TM after rigorous adjustment for confounders. Individuals in the upper tertile of baseline BRI encountered 1.14-fold (HR = 1.14, 95% CI 1.01–1.29; *p* = 0.0413) and 1.27-fold (HR = 1.27, 95% CI 1.12–1.43; *p* < 0.0001) increased risks of MACEs and TM, respectively, relative to those in the lower tertile. A marked trend was observed, associating elevated BRI with greater risks of MACEs (*p* = 0.0422) and TM (*p* < 0.0001). Furthermore, a single SD increment in BRI was significantly correlated with a 6% rise in MACE risk (*p* = 0.0265) and an 11% surge in TM (*p* < 0.0001). In contrast, after undergoing the same multivariate regression analysis, BMI was found to be associated with TM, with the highest baseline BMI quartile showing a 1.19-fold higher risk of mortality compared to the lowest quartile (HR = 1.19, 95% CI 1.06–1.35, *p* = 0.0035). However, no significant association was found between the highest and lowest baseline BMI quartiles and MACEs (HR = 1.05, 95% CI 0.93–1.19, *p* = 0.4647). Notably, the associations of BRI with MACEs and TM were comparable to those observed for the WHtR and the conicity index. In summary, although BMI correlated with TM among patients with T2DM, it did not notably affect the aggregate risk of MACEs in this investigation.

**Table 2:** Risk of MACEs and total mortality based on BRI, BMI, WHtR and conicity index.

Outcome	Events/n	Non-adjusted		Model 1		Model 2		Model 3	
		HR (95%CI)	*p* value	HR (95%CI)	*p* value	HR (95%CI)	*p* value	HR (95%CI)	*p* value
MACEs									
BRI		1.04 (1.02, 1.07)	0.0012	1.04 (1.01, 1.07)	0.0023	1.04 (1.01, 1.07)	0.0028	1.03 (1.00, 1.06)	0.0265
Tertile 1	550/3382	Ref		Ref		Ref		Ref	
Tertile 2	617/3380	1.15 (1.02, 1.29)	0.0203	1.14 (1.01, 1.28)	0.0310	1.13 (1.00, 1.27)	0.0464	1.10 (0.97, 1.24)	0.1266
Tertile 3	635/3386	1.19 (1.06, 1.34)	0.0026	1.19 (1.06, 1.34)	0.0039	1.19 (1.05, 1.34)	0.0056	1.14 (1.01, 1.29)	0.0413
Per 1 SD		1.08 (1.03, 1.13)	0.0012	1.08 (1.03, 1.13)	0.0023	1.08 (1.03, 1.13)	0.0028	1.06 (1.01, 1.11)	0.0265
P for trend			0.0027		0.0039		0.0057		0.0422
BMI		1.00 (0.99, 1.01)	0.5378	1.01 (1.00, 1.02)	0.0753	1.01 (1.00, 1.02)	0.0788	1.00 (0.99, 1.01)	0.3858
Tertile 1	606/3382	Ref		Ref		Ref		Ref	
Tertile 2	575/3382	0.95 (0.85, 1.06)	0.3788	0.95 (0.84, 1.07)	0.3665	0.96 (0.85, 1.07)	0.4480	0.93 (0.83, 1.05)	0.2540
Tertile 3	620/3382	1.03 (0.92, 1.16)	0.5637	1.10 (0.98, 1.24)	0.1020	1.10 (0.98, 1.25)	0.1040	1.05 (0.93, 1.19)	0.4647
Per 1 SD		1.01 (0.97, 1.06)	0.53781	1.05 (1.00, 1.10)	0.0753	1.05 (0.99, 1.10)	0.0788	1.02 (0.97, 1.08)	0.3858
P for trend			0.5621		0.1067		0.1065		0.4641
WHtR		2.63 (1.46, 4.71)	0.0012	2.56 (1.38, 4.77)	0.0030	2.57 (1.36, 4.84)	0.0035	2.04 (1.06, 3.92)	0.0324
Tertile 1	550/3381	Ref		Ref		Ref		Ref	
Tertile 2	617/3381	1.14 (1.02, 1.28)	0.0211	1.15 (1.02, 1.29)	0.0214	1.13 (1.00, 1.27)	0.0475	1.10 (0.97, 1.23)	0.1295
Tertile 3	635/3386	1.19 (1.06, 1.34)	0.0027	1.20 (1.06, 1.35)	0.0033	1.19 (1.05, 1.34)	0.0057	1.14 (1.00, 1.29)	0.0419
Per 1 SD		1.08 (1.03, 1.13)	0.0012	1.08 (1.03, 1.13)	0.0030	1.08 (1.02, 1.13)	0.0035	1.06 (1.00, 1.11)	0.0324
P for trend			0.0028		0.0033		0.0057		0.0428
Conicity index		5.94 (3.56, 9.93)	<0.0001	2.39 (1.40, 4.10)	0.0014	2.34 (1.36, 4.03)	0.0023	2.10 (1.21, 3.64)	0.0081
Tertile 1	515/3382	Ref		Ref		Ref		Ref	
Tertile 2	582/3382	1.17 (1.04, 1.32)	0.0095	1.06 (0.94, 1.19)	0.3822	1.06 (0.94, 1.20)	0.3278	1.05 (0.93, 1.18)	0.4513
Tertile 3	704/3382	1.46 (1.30, 1.63)	<0.0001	1.20 (1.06, 1.34)	0.0028	1.19 (1.06, 1.34)	0.0036	1.17 (1.04, 1.32)	0.0105
Per 1 SD		1.17 (1.12, 1.23)	<0.0001	1.08 (1.03, 1.14)	0.0014	1.08 (1.03, 1.13)	0.0023	1.07 (1.02, 1.12)	0.0081
P for trend			<0.0001		0.0023		0.0030		0.0088
Total mortality									
BRI		1.06 (1.04, 1.08)	<0.0001	1.07 (1.04, 1.09)	<0.0001	1.07 (1.04, 1.09)	<0.0001	1.06 (1.03, 1.08)	<0.0001
Tertile 1	577/3382	Ref		Ref		Ref		Ref	
Tertile 2	635/3380	1.12 (1.00, 1.25)	0.0526	1.10 (0.98, 1.23)	0.1068	1.10 (0.98, 1.23)	0.1216	1.06 (0.95, 1.20)	0.2970
Tertile 3	714/3386	1.29 (1.16, 1.44)	<0.0001	1.33 (1.19, 1.50)	<0.0001	1.33 (1.18, 1.49)	<0.0001	1.27 (1.12, 1.43)	<0.0001
Per 1 SD		1.12 (1.07, 1.17)	<0.0001	1.13 (1.08, 1.19)	<0.0001	1.13 (1.08, 1.19)	0.0003	1.11 (1.06, 1.17)	<0.0001
P for trend			<0.0001		<0.0001		<0.0001		<0.0001
BMI		1.00 (1.00, 1.01)	0.3800	1.02 (1.01, 1.03)	0.0001	1.02 (1.01, 1.03)	<0.0001	1.01 (1.00, 1.02)	0.0038
Tertile 1	649/3382	Ref		Ref		Ref		Ref	
Tertile 2	605/3382	0.94 (0.85, 1.06)	0.3151	0.98 (0.88, 1.10)	0.7834	1.00 (0.89, 1.12)	0.9880	0.97 (0.87, 1.09)	0.6643
Tertile 3	671/3382	1.07 (0.96, 1.19)	0.2299	1.25 (1.12, 1.40)	0.0001	1.28 (1.14, 1.43)	<0.0001	1.19 (1.06, 1.35)	0.0035
Per 1 SD		1.02 (0.98, 1.07)	0.3800	1.10 (1.05, 1.16)	0.0001	1.11 (1.06, 1.17)	<0.0001	1.11 (1.06, 1.17)	<0.0001
P for trend			0.2298		0.0002		<0.0001		0.0040
WHtR		4.12 (2.35, 7.23)	<0.0001	5.09 (2.79, 9.29)	<0.0001	4.83 (2.62, 8.91)	<0.0001	3.73 (1.99, 6.99)	<0.0001
Tertile 1	577/3381	Ref		Ref		Ref		Ref	
Tertile 2	635/3381	1.12 (1.00, 1.25)	0.0542	1.11 (0.99, 1.24)	0.0845	1.09 (0.98, 1.23)	0.1245	1.06 (0.95, 1.19)	0.3032
Tertile 3	714/3386	1.29 (1.16, 1.44)	<0.0001	1.34 (1.20, 1.51)	<0.0001	1.33 (1.18, 1.49)	<0.0001	1.27 (1.12, 1.43)	0.0001
Per 1 SD		1.12 (1.07, 1.17)	<0.0001	1.14 (1.08, 1.19)	<0.0001	1.13 (1.08, 1.19)	<0.0001	1.11 (1.06, 1.17)	<0.0001
P for trend			<0.0001		<0.0001		<0.0001		<0.0001
Conicity index		12.73 (7.76, 20.87)	<0.0001	4.06 (2.42, 6.82)	<0.0001	3.57 (2.12, 6.04)	<0.0001	3.30 (1.95, 5.61)	<0.0001
Tertile 1	513/3382	Ref		Ref		Ref		Ref	
Tertile 2	613/3382	1.22 (1.08, 1.37)	0.0009	1.05 (0.93, 1.19)	0.3961	1.05 (0.93, 1.18)	0.4517	1.04 (0.92, 1.17)	0.5392
Tertile 3	799/3382	1.65 (1.48, 1.85)	<0.0001	1.28 (1.14, 1.44)	<0.0001	1.26 (1.12, 1.41)	<0.0001	1.24 (1.10, 1.39)	0.0004
Per 1 SD		1.26 (1.20, 1.31)	<0.0001	1.13 (1.08, 1.19)	<0.0001	1.12 (1.07, 1.18)	<0.0001	1.11 (1.06, 1.17)	<0.0001
P for trend			<0.0001		<0.0001		<0.0001		0.0002

Data are expressed as HR and 95% CIs (reported in parentheses) as assessed by multivariable Cox regression analysis.

Model 1: adjusted for sex, race, age, education, living situation, CVD history, duration of diabetes, previous hypertension, previous hyperlipidemia, proteinuria, heart failure, smoking, and depression.

Model 2: adjusted for model 1 covariables plus SBP, DBP, FPG, HbA1c, TC, TG, LDL-C, HDL-C and eGFR.

Model 3: adjusted for model 2 covariables plus the medications use, diuretics, CCBs, beta-blockers, biguanides, meglitinides, thiazolidinediones, insulins, cholesterol absorption inhibitors and aspirin.

BRI, body roundness index; BMI, body mass index; WHtR, waist-to-height ratio; MACEs, major adverse cardiovascular events; HR, hazard ratio; CI, confidence interval.

### Detection of nonlinear relationships

**Figure 3:**
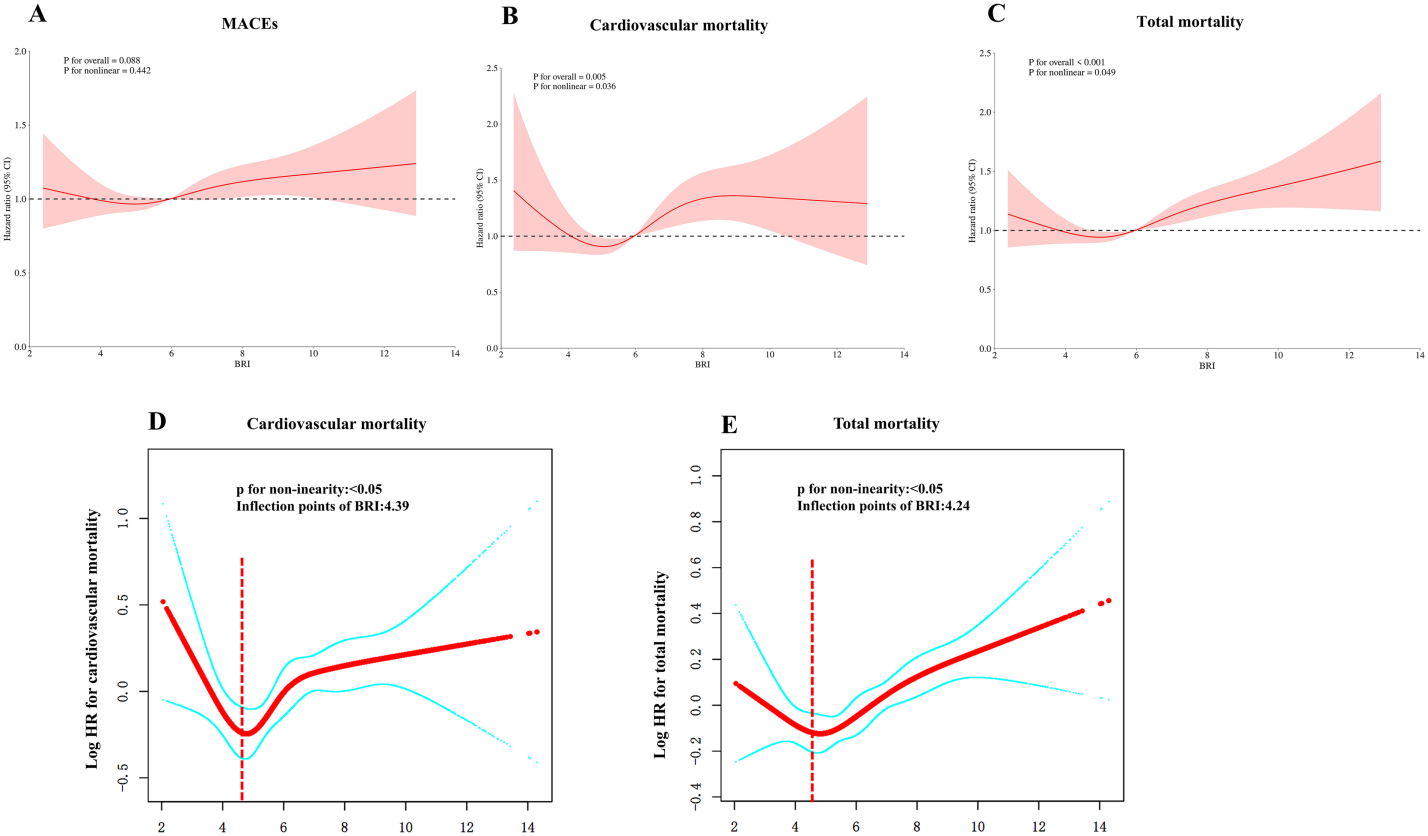
Restricted cubic spline and smooth curve fitting analyses were performed to assess the relationship between BRI and the risk of cardiovascular and total mortality. **(A)** MACEs; **(B)** cardiovascular mortality; **(C)** total mortality; **(D)** cardiovascular mortality; **(E)**, total mortality. The hazard ratios shown are adjusted for Model 3, including sex, race, age, education, living situation, CVD history, duration of diabetes, previous hypertension, previous hyperlipidemia, proteinuria, heart failure, smoking, depression, SBP, DBP, FPG, HbA1c, TG, LDL-C, HDL-C, eGFR, diuretics, CCBs, beta-blockers, biguanides, meglitinides, thiazolidinediones, insulins, cholesterol absorption inhibitors, and aspirin.

To probe potential nonlinear dynamics, RCS and smooth curve fitting were deployed to examine the relationship between BRI, MACEs, and TM. A linear association was confirmed between BRI and MACEs, while nonlinear associations were identified concerning BRI with cardiovascular and TM. These results were corroborated by log-likelihood tests (*p* < 0.05). Inflection points were established at 4.39 for cardiovascular mortality and 4.24 for TM through a recursive approach (Figure 3). A two-stage Cox proportional hazards model was implemented to calculate the effect sizes and confidence intervals around these points. Beyond these inflection points, every unit decrement in BRI translated into a 9% decrease in the probability of cardiovascular mortality (HR = 1.09, 95% CI: 1.04–1.14) (Table 3). Similarly, for TM, each unit decrease in BRI past the inflection point resulted in a 9% reduction in mortality risk (HR = 1.09, 95% CI: 1.04–1.14).

**Table 3:** Analysis of the threshold effects of BRI levels on cardiovascular and total mortality.

Outcomes	One linear-regression model	Inflection point (K)	<K, effect 1	>K, effect 2	*p* value for LRT
Cardiovascular mortality	1.06 (1.01, 1.10) 0.0110	4.39	0.76 (0.58,1.00) *p* = 0.052	1.09 (1.04,1.14) *p* = 0.0008	0.023
Total mortality	1.06 (1.03, 1.08) < 0.0001	4.24	0.84 (0.70,1.01) *p* = 0.0679	1.07 (1.04,1.10) *p* < 0.0001	0.018

The effects shown are adjusted for model 3, including sex, race, age, education, living situation, CVD history, duration of diabetes, previous hypertension, previous hyperlipidemia, proteinuria, heart failure, smoking, depression, SBP, DBP, FPG, HbA1c, TG, LDL-C, HDL-C, eGFR, diuretics, CCBs, beta-blockers, biguanides, meglitinides, thiazolidinediones, insulins, cholesterol absorption inhibitors and aspirin.

BRI, body roundness index; LRT, likelihood ratio test.

### Subgroup and interaction analysis

**Figure 4:**
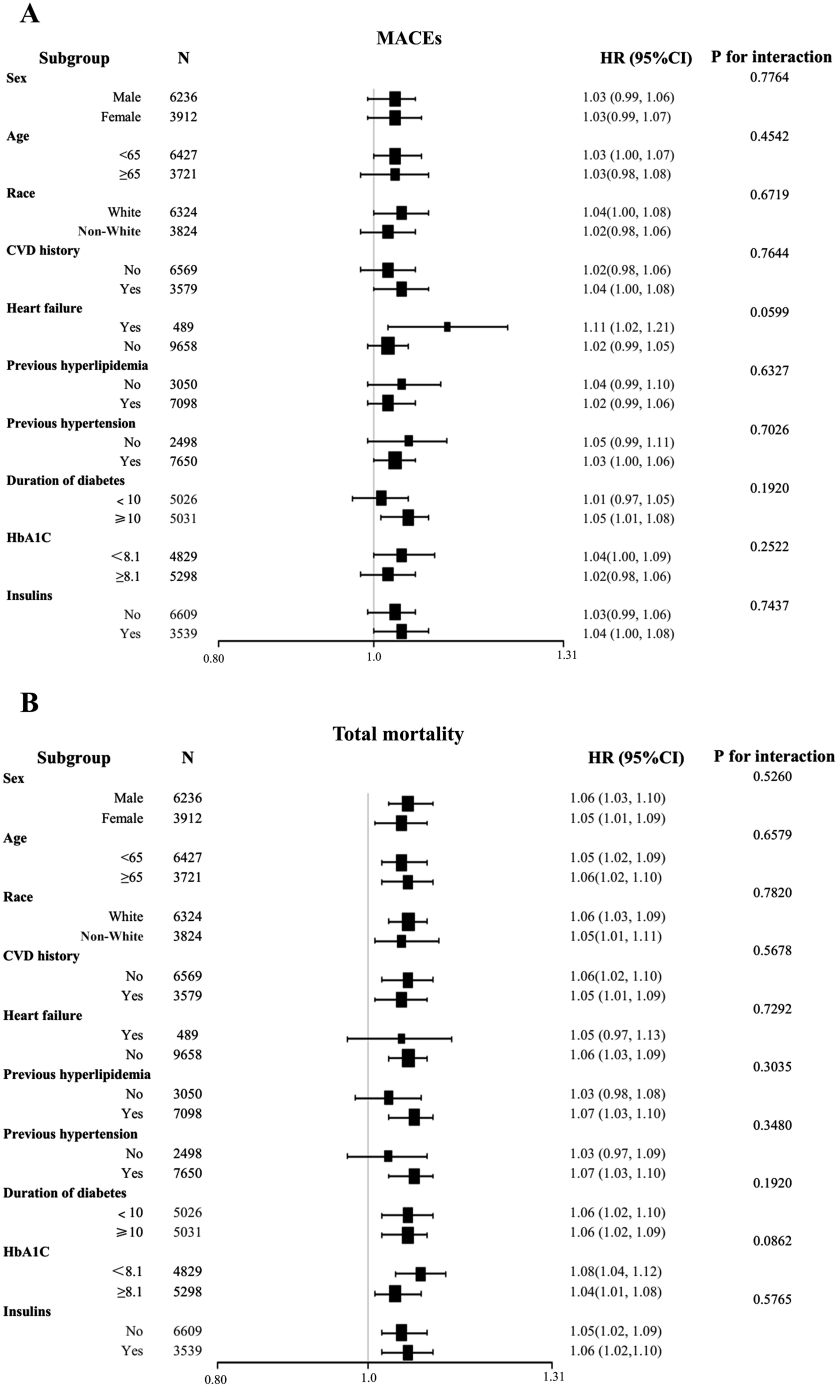
Subgroup and interaction analyses were conducted to examine the relationship between BRI and the risks of MACEs and total mortality. **(A)** MACEs; **(B)** total mortality. BRI, body roundness index; MACEs, major adverse cardiovascular events; HR, hazard ratio; CI, confidence interval.

To further delineate BRI’s role across various demographics and conditions, subgroup and interaction analyses were conducted (Figure 4). These analyses, stratified by age, gender, ethnicity, cardiovascular history, heart failure, hyperlipidemia, hypertension, diabetes duration, HbA1c levels, and insulin treatment, showed consistent associations across all categories. No significant interactions were observed between baseline BRI and these variables (P for interaction > 0.05).

### Sensitivity analysis

Robustness checks via sensitivity analysis utilized multiple imputation to mitigate the effects of incomplete data, applying multivariate Cox proportional hazards regression to the imputed dataset. These analyses corroborated the findings, aligning with those obtained through the deletion method (Supplementary Table 3), further substantiating the study’s conclusions.

## Discussion

In this study, we explored associations between BRI and both MACEs and TM in patients with T2DM within the ACCORD/ACCORDION cohorts. Our findings demonstrate that elevated BRI is an independent risk factor for MACEs and TM in T2DM patients. Incorporating BRI into clinical assessment may therefore enhance the prevention and management of cardiovascular complications in this high-risk population.

CVD remains a formidable global health challenge, imposing significant burdens on healthcare systems and families internationally (29, 30). Being an independent risk factor, diabetes is closely linked with adverse outcomes, with a significant number of patients succumbing to cardiovascular complications (31, 32).

Consequently, there is a pressing requirement to develop innovative modulated indicators that can more precisely forecast the risk of adverse cardiovascular events, thereby enabling the creation of tailored prevention strategies for patients with T2DM. Although BMI is universally acknowledged as a key measure of obesity and has been the subject of extensive research, recent studies have pointed out a discrepancy in the way BMI gauges both obesity and mortality risk across different populations (33, 34). Epidemiological research has indicated the presence of abdominal obesity in individuals within a normal BMI range (35, 36). Conversely, an elevated waist circumference (WC) is typically indicative of increased fat accumulation, offering a more accurate representation of the actual burden of central obesity (37). However, the fundamental flaw of WC measurements lies in their disregard for height variations, potentially causing misestimations of abdominal obesity in individuals of varying stature (38). The BRI, integrating weight and waist measurements, offers a superior estimation of body fat and visceral adipose tissue proportions compared to BMI (16). The association of BRI with CVD risk is multifactorial: primarily, obesity is a known risk factor for CVD, and BRI closely associates with prevalent CVD risk factors such as hypertension and diabetes; secondarily, adipose tissue is likely to amplify pro-inflammatory cytokines, boost oxidative stress, and trigger neurohormones, collectively escalating cardiometabolic risk and precipitating left ventricular remodeling (39, 40). Moreover, obesity can directly compromise myocardial integrity, adversely affecting cardiac structure and function, potentially culminating in obesity-associated cardiomyopathy or sudden death (41, 42). Prior research predominantly explored BRI’s role in forecasting CVD incidence or cardiometabolic risks (24, 43–45), with scant studies investigating BRI’s correlation with CVD or TM. Zhang et al. recently observed (22) an increase in BRI among American adults over two decades, showing a U-shaped correlation with TM. Yang et al. confirmed (21) BRI’s independent association with mortality from cardiovascular conditions and TM in hypertensive subjects. A recent study reported that BRI is a valuable predictive indicator for all-cause and cardiovascular mortality in patients with diabetes and pre-diabetes, with a U-shaped association observed for both (23). Yet, the interaction between BRI and MACEs or TM within the ACCORD/ACCORDION cohort remains unexplored, with no inquiries into the association between BRI and the risk of MACEs. Our investigation has broadened and refined the scope of earlier studies.

This investigation marks the inaugural linkage between the BRI and increased risk of MACEs as well as TM in patients with T2DM participating in the ACCORD study. Within this cohort of 10,148 patients with T2DM, our analysis revealed that elevated baseline BRI levels were associated with greater risks of forthcoming MACEs and TM. Notably, this association persisted as statistically significant even after adjusting for primary confounders. Comparatively, patients in the highest baseline BRI tertile faced risks of MACEs and TM that were 1.14 and 1.27 times greater, respectively, than those in the lowest tertile. Furthermore, we investigated the relationship between BMI and health outcomes. Although BMI correlated with TM in patients with T2DM, elevated baseline BMI did not significantly associate with the risk of future MACEs in our findings. To characterize the shape of the associations, we modeled them using Cox proportional hazards regression with smooth curve fitting, which can flexibly capture nonlinear dynamics. Our results identified a linear association between BRI and MACEs, but suggested a potentially non-linear correlation between BRI and the risks for cardiovascular mortality and TM, with critical inflection points occurring at 4.39 and 4.24, respectively. These findings carry substantial clinical importance, potentially enhancing patient consultations and enabling better patient self-management and monitoring, thereby laying a groundwork for public health initiatives aimed at mitigating cardiovascular and TM risks in patients with T2DM. By sustaining a BRI below 4.24 through dietary and lifestyle adjustments, the probability of cardiovascular and TM decreases markedly. Results from additional stratification and interaction analyses aligned closely with the main outcomes. To ensure the reliability of our findings, a sensitivity analysis was also performed.

Our results suggest that the BRI may serve as a robust indicator for the risk of MACEs and TM among patients with T2DM. By maintaining a healthy BRI level, there is a significant reduction in the risk of future MACEs and TM. Thus, regular monitoring of BRI during the initial clinical assessment, ongoing treatment, and extensive management of diabetes, combined with effective body type management, is essential for reducing MACEs incidence and mortality rates in patients with T2DM. In addition, compared to BMI, BRI showed stronger associations with the risk of MACEs.

Our study is the first to establish the BRI as a significant risk factor of MACEs and TM in a T2DM population within the ACCORD/ACCORDION study. This finding is supported by a long-term, large-scale cohort study design and rigorous statistical control for confounding variables, confirming the clinical relevance of BRI. Beyond its independent association, the BRI may serve as a complementary measure to classical cardiovascular risk factors, potentially improving risk stratification and patient counseling in clinical practice.

In this study, BRI showed stronger associations with MACEs and total mortality than BMI, while its associations were comparable to those of WHtR and the conicity index. These findings suggest that body shape–based indices may capture cardiovascular risk more accurately than measures of overall obesity. Recent studies have also highlighted the importance of WHtR in assessing obesity-related risk, and have underscored the diagnostic value of combining BMI and WHtR for classifying obesity, highlighting the complementary roles of overall and central adiposity (46–48). Building on this concept, integrating BRI with WHtR may provide a more practical and comprehensive tool for clinical assessment of obesity-related cardiovascular risk and for improving patient-centered communication. Future research should validate these combined indices and clarify their potential utility in risk stratification and clinical decision-making.

Nonetheless, the research has certain limitations. Primarily, it constitutes a *post hoc* analysis within the ACCORD and ACCORDION trials. Although we adjusted for known confounders in our multivariate Cox regression analysis, the possibility of residual or unmeasured confounders, such as enduring changes in lifestyle and dietary habits, persists. Secondly, due to its observational design, this study cannot confirm a causal link between baseline BRI and future risks of MACEs and TM mortality among patients with T2DM. Therefore, rigorously designed prospective intervention studies are required to substantiate these findings further. Thirdly, since the trial participants were primarily high-risk cardiovascular patients with T2DM from North America, extrapolating these results to different populations should be approached with caution. Variabilities in metabolic profiles, lipid concentrations, and cardiovascular risks among different ethnic groups necessitate additional research across diverse cohorts to verify these findings. Future research should include external, multi-ethnic validation and head-to-head comparisons with other central adiposity indices before clinical application. In addition, although the ACCORD/ACCORDION data were collected up to 2014, the study included a median follow-up of 8.82 years and remains a classic, well-characterized cohort in diabetes research. Given the long-term and biologically stable nature of the associations between anthropometric indices such as the BRI and cardiovascular outcomes, our findings are likely to remain robust and relevant. Lastly, excluding participants with incomplete baseline data might introduce selection bias, thereby impacting the generalizability of the results. Although we employed multiple imputation to manage missing data and found consistency with deletion analysis outcomes, future studies should aim for more comprehensive data collection to better generalize the findings.

In summary, this study is the inaugural comparative analysis of BRI and BMI regarding their ability to predict future MACEs and TM risks in patients with T2DM, affirming the superiority of BRI. We discovered that BRI independently associates with future MACEs and TM risks in these patients. Thus, BRI constitutes an effective and significant predictor, offering a scientific foundation for its application as a straightforward, non-invasive clinical screening instrument. This could significantly improve personalized care and public health interventions for patients with T2DM.

## Data Availability

The original contributions presented in the study are included in the article/Supplementary material, further inquiries can be directed to the corresponding author.

## Glossary

BRI: Body roundness index
BMI: Body mass index
WHtR: Waist-to-height ratio
MACEs: Major adverse cardiovascular events
TM: Total mortality
T2DM: Type 2 diabetes mellitus
GED: General education development
CVD: Cardiovascular disease
SBP: Systolic blood pressure
DBP: Diastolic blood pressure
FPG: Fasting plasma glucose
HbA1C: Glycated hemoglobin
TC: Total cholesterol
TG: Triglycerides
LDL-C: Low-density lipoprotein cholesterol
HDL-C: High-density lipoprotein cholesterol
eGFR: Estimated glomerular filtration rate
ARB: Angiotensin receptor blocker
ACEI: Angiotensin converting enzyme inhibitors
CCB: Calcium channel blockers
ACCORD: Action to control cardiovascular risk in diabetes
ACCORDION: The ACCORD follow-up study
ANOVA: Analysis of variance
WC: Waist circumference
HR: Hazard ratio
CI: Confidence interval
RCS: Restricted cubic spline
AUC: Area under curve
NRI: Net reclassification index
IDI: Integrated discrimination improvement
VIF: Variance inflation factor
