# Supplementary material for: BRI shows stronger association than BMI for MACE in patients with T2DM: insights from the ACCORD study
Source: Front Nutr. 2025 Dec 12;12:1720948. doi: 10.3389/fnut.2025.1720948 (PMC12742206; doi:10.3389/fnut.2025.1720948)
Supplement: Supplementary file 1 [file Table_1.docx]

**Supplemental Files**

**Supplemental Table 1.** Univariate analysis for predictin MACEs.

**Supplemental Table 2.**Assessment of multicollinearity among adjustment variables.

**Supplemental Table 3.** Risk of MACEs and total mortality based on BRI and BMI after employing multiple imputation by chained equations to address missing BRI and BMI values at baseline.

**Supplemental Table 1.** **Univariate analysis for predictin MACEs.**

|  | **HR** | **95% CI** | ***p* value** |
| --- | --- | --- | --- |
| the BRI tertile |  |  |  |
| Tertile 1 | 1.00 |  |  |
| Tertile 2 | 1.19 | 1.06-1.33 | 0.0036 |
| Tertile 3 | 1.19 | 1.06-1.34 | 0.0024 |
| Age (years) | 1.04 | 1.03-1.04 | <0.0001 |
| Male | 1.45 | 1.32-1.59 | <0.0001 |
| White | 1.35 | 1.22-1.49 | <0.0001 |
| Education level |  |  |  |
| Less than high school | 1.00 |  |  |
| High school graduate or GED | 0.83 | 0.72-0.96 | 0.0131 |
| Some college | 0.86 | 0.75-0.98 | 0.0271 |
| College degree or higher | 0.68 | 0.59-0.79 | <0.0001 |
| Living alone | 1.11 | 0.99-1.25 | 0.0631 |
| Depression | 1.12 | 1.12-1.39 | <0.0001 |
| smoking | 1.32 | 1.19-1.45 | <0.0001 |
| Alcohol | 0.92 | 0.82-1.02 | 0.1118 |
| Proteinuria | 1.30 | 1.16-1.45 | <0.0001 |
| eGFR | 0.99 | 0.99-1.00 | <0.0001 |
| Duration of diabetes (years) | 1.02 | 1.02-1.03 | <0.0001 |
| CVD history | 2.39 | 2.18-2.63 | <0.0001 |
| Previous heart failure | 2.44 | 2.08-2.86 | <0.0001 |
| Previous hypertension | 1.21 | 1.08-1.35 | 0.0009 |
| Previous hyperlipidemia | 1.09 | 0.98-1.21 | 0.1060 |
| Blood pressure (mmHg) |  |  |  |
| SBP | 1.01 | 1.00-1.01 | <0.0001 |
| DBP | 0.99 | 0.98-0.99 | <0.0001 |
| Heart rate (bpm) | 1.00 | 0.99-1.00 | 0.2358 |
| HbA1c (%) | 1.12 | 1.08-1.17 | <0.0001 |
| FPG (mmol/L)  BMI (kg/m^2^) | 1.00  1.00 | 1.00-1.00  0.99-1.01 | <0.0001  0.5378 |
| TG (mg/dL)  TC (mg/dL)  LDL-C (mg/dL)  HDL(mg/dL | 1.00  1.00  1.00  0.98 | 1.00-1.00  1.00-1.00  1.00-1.00  0.98-0.99 | 0.0001  0.0531  0.0334  <0.0001 |
| Medications use  Diuretics | 1.33 | 1.21-1.46 | <0.0001 |
| Insulin | 1.45 | 1.32-1.59 | <0.0001 |
| ARB/ACEI | 1.01 | 0.92-1.12 | 0.7935 |
| CCB | 1.40 | 1.26-1.57 | <0.0001 |
| Beta blockers  Biguanides | 1.68  0.79 | 1.53-1.85  0.72-0.87 | <0.0001  <0.0001 |
| Thiazolidinediones  Sulfonylureas  Meglitinides | 0.82  0.92  0.84 | 0.73-0.92  0.84-1.01  0.61-1.15 | 0.0009  0.0733  0.2720 |
| Aspirin | 1.12 | 1.02-1.23 | 0.0189 |
| Statins | 1.09 | 0.99-1.20 | 0.0792 |
| Cholesterol absorption inhibitors | 1.18 | 0.86-1.63 | 0.3133 |

Abbreviations:HR, hazard ratio; CI, confidence interval; BRI, body roundness index; GED, general education development; CVD, cardiovascular disease; bpm, beet per minute; HbA1c, hemoglobin A1c; FPG, fasting plasma glucose; TG, triglycerides; eGFR, estimated glomerular filtration rate; BMI, body mass index; SBP, systolic blood pressure; DBP, diastolic blood pressure; ACEI, angiotensin-converting enzyme inhibitors; ARB, angiotensin receptor blockers; CCB, calcium channel blockers; MACEs, major adverse cardiovascular events.

**Supplemental Table 2.Assessment of multicollinearity among adjustment variables.**

| **Variables** | **Tolerance** |  | **VIF** |
| --- | --- | --- | --- |
| BRI | 0.860 |  | 1.163 |
| Sex | 0.718 |  | 1.392 |
| Race | 0.837 |  | 1.194 |
| Age | 0.729 |  | 1.371 |
| Livealon | 0.960 |  | 1.042 |
| Education | 0.925 |  | 1.081 |
| Duration of diabetes | 0.753 |  | 1.329 |
| History of cardiovascular disease | 0.771 |  | 1.297 |
| Previous hypertension | 0.856 |  | 1.169 |
| Previous hyperlipidemia | 0.898 |  | 1.114 |
| Proteinuria | 0.963 |  | 1.039 |
| Heart failure | 0.930 |  | 1.075 |
| Depression | 0.923 |  | 1.083 |
| Smoking status | 0.899 |  | 1.112 |
| SBP | 0.609 |  | 1.643 |
| DBP | 0.554 |  | 1.806 |
| FPG | 0.718 |  | 1.393 |
| HbA1c | 0.736 |  | 1.359 |
| triglycerides | 0.775 |  | 1.290 |
| LDL-C | 0.891 |  | 1.290 |
| HDL-C | 0.687 |  | 1.456 |
| eGFR | 0.880 |  | 1.136 |
| Diuretics | 0.865 |  | 1.156 |
| CCB | 0.941 |  | 1.063 |
| Beta-blockers | 0.808 |  | 1.238 |
| Biguanides | 0.893 |  | 1.120 |
| Meglitinide | 0.993 |  | 1.007 |
| Thiazolidinediones | 0.941 |  | 1.063 |
| Insulins | 0.733 |  | 1.364 |
| Aspirin | 0.927 |  | 1.079 |
| Cholesterol absorption inhibitors | 0.989 |  | 1.011 |

BRI, body roundness index;VIF, Variance inflation factor; SBP, systolic blood pressure; DBP, diastolic blood pressure; FPG, fasting blood glucose; eGFR, estimated glomerular filtration rate; CCB, Calcium Channel Blockers.

**Supplemental Table 3. Risk of MACEs and total mortality based on BRI and BMI after employing multiple imputation by chained equations to address missing BRI and BMI values at baseline.**

| **Outcome** | **Non-adjusted** | | **Model 1** | | **Model 2** | | **Model 3** | |
| --- | --- | --- | --- | --- | --- | --- | --- | --- |
|  | **HR (95%CI)** | ***P* value** | **HR (95%CI)** | ***P* value** | **HR (95%CI)** | ***P* value** | **HR (95%CI)** | ***P* value** |
| **MACEs** |  |  |  |  |  |  |  |  |
| **BRI** | 1.04 (1.02, 1.07) | 0.0013 | 1.04 (1.02, 1.07) | 0.0013 | 1.04 (1.01, 1.07) | 0.0077 | 1.03 (1.00, 1.06) | 0.0468 |
| Tertile1 | Ref |  | Ref |  | Ref |  | Ref |  |
| Tertile2 | 1.15(1.03,1.29) | 0.0155 | 1.14 (1.02, 1.28) | 0.0253 | 1.13 (1.01, 1.27) | 0.0382 | 1.10 (0.98, 1.24) | 0.1123 |
| Tertile3 | 1.20(1.07,1.34) | 0.0015 | 1.20(1.07, 1.35) | 0.0024 | 1.19 (1.06, 1.35) | 0.0042 | 1.15 (1.01, 1.30) | 0.0287 |
| Per 1 SD | 1.08(1.03,1.13) | 0.0011 | 1.08 (1.03, 1.13) | 0.0011 | 1.08 (1.03, 1.13) | 0.0011 | 1.06 (1.01, 1.12) | 0.0246 |
| P for trend |  | 0.0035 |  | 0.016 |  | 0.0045 |  | 0.0285 |
| **BMI** | 1.00(0.99,1.01) | 1.0000 | 1.01 (1.00, 1.02) | 0.0489 | 1.01 (1.00, 1.02) | 0.0489 | 1.00 (0.99, 1.01) | 1.0000 |
| Tertile1 | Ref |  | Ref |  | Ref |  | Ref |  |
| Tertile2 | 0.94(0.84,1.06) | 0.3337 | 0.95 (0.85, 1.07) | 0.3874 | 0.95 (0.85, 1.07) | 0.3824 | 0.93 (0.83, 1.05) | 0.2263 |
| Tertile3 | 1.03(0.92,1.15) | 0.6036 | 1.10 (0.98, 1.24) | 0.1123 | 1.10 (0.98, 1.24) | 0.1123 | 1.05 (0.93, 1.19) | 0.4422 |
| Per 1 SD | 1.01(0.97,1.06) | 0.6602 | 1.05 (1.00, 1.10) | 0.0448 | 1.05 (1.00, 1.10) | 0.0448 | 1.02 (0.97, 1.08) | 0.4699 |
| P for trend |  | 0.4743 |  | 0.1053 |  | 0.1211 |  | 0.5410 |
| **Total mortality** |  |  |  |  |  |  |  |  |
| **BRI** | 1.06 (1.04, 1.08) | <0.0001 | 1.07 (1.05, 1.10) | <0.0001 | 1.07 (1.05, 1.10) | <0.0001 | 1.05 (1.03, 1.08) | 0.0001 |
| Tertile1 | Ref |  | Ref |  | Ref |  | Ref |  |
| Tertile2 | 1.11 (0.99, 1.24) | 0.0648 | 1.09 (0.97, 1.22) | 0.1372 | 1.08 (0.97, 1.21) | 0.1768 | 1.05 (0.94, 1.18) | 0.3871 |
| Tertile3 | 1.29 (1.16, 1.44) | <0.0001 | 1.33 (1.19, 1.49) | <0.0001 | 1.32 (1.18, 1.48) | <0.0001 | 1.26 (1.12, 1.42) | 0.0001 |
| Per 1 SD | 1.12 (1.07, 1.17) | <0.0001 | 1.13 (1.08, 1.22) | <0.0001 | 1.13 (1.08, 1.18) | <0.0001 | 1.11 (1.06, 1.16) | <0.0001 |
| P for trend |  | <0.0001 |  | <0.0001 |  | <0.0001 |  | 0.0001 |
| **BMI** | 1.00 (1.00, 1.00) | 1.0000 | 1.02 (1.01, 1.03) | 0.0001 | 1.02 (1.01, 1.03) | 0.0001 | 1.01 (1.00, 1.02) | 0.0489 |
| Tertile1 | Ref |  | Ref |  | Ref |  | Ref |  |
| Tertile2 | 0.95 (0.85, 1.06) | 0.3625 | 0.99 (0.88, 1.11) | 0.8627 | 1.01 (0.90, 1.13) | 0.8639 | 0.98 (0.88, 1.10) | 0.7227 |
| Tertile3 | 1.07 (0.97, 1.20) | 0.1904 | 1.26 (1.12, 1.41) | <0.0001 | 1.28 (1.14, 1.44) | <0.0001 | 1.20 (1.07, 1.35) | 0.0021 |
| Per 1 SD | 1.02 (0.98, 1.07) | 0.3770 | 1.10 (1.05, 1.16) | <0.0001 | 1.11 (1.06, 1.17) | <0.0001 | 1.08 (1.03, 1.14) | 0.0029 |
| P for trend |  | 0.1832 |  | 0.0001 |  | <0.0001 |  | 0.0017 |

Data are expressed as HR and 95% CIs (reported in parentheses) as assessed by multivariable Cox regression analysis.

Model 1: adjusted for sex, race, age, education, living situation, CVD history, duration of diabetes, previous hypertension ,previous hyperlipidemia, proteinuria, heart failure, smoking, and depression.

Model 2: adjusted for model 1 covariables plus SBP, DBP, FPG, HbA1c, TC, TG, LDL-C, HDL-C and eGFR.

Model 3: adjusted for model 2 covariables plus the medications use, diuretics, CCBs, beta-blockers, biguanides, meglitinides, thiazolidinediones, insulins, cholesterol absorption inhibitors and aspirin.

Abbreviations: BRI, body roundness index; BMI, body mass index; MACEs, major adverse cardiovascular events; HR,hazard ratio; CI: confidence interval.
